# Supplementary material for: Emotion recognition while applying cosmetic cream using deep learning from EEG data; cross-subject analysis
Source: PLoS One. 2022 Nov 10;17(11):e0274203. doi: 10.1371/journal.pone.0274203 (PMC9648831; doi:10.1371/journal.pone.0274203)
Supplement: Attachment — Submitted filename: Response to Reviewers..docx [file pone.0274203.s001.docx]

Dear Anwar P.P. Abdul Majeed.

Thank you for giving me the opportunity to submit a revised draft of my manuscript titled “Emotion recognition while applying cosmetic cream using deep learning from EEG data; cross-subject analysis” PLOS ONE. We appreciate the time and effort that you and the reviewers have dedicated to providing your valuable feedback on my manuscript. We are grateful to the reviewers for their insightful comments on my paper. We have been able to incorporate changes to reflect most of the suggestions provided by the reviewers. We have highlighted the changes within the manuscript. Here is a point-by-point response to the reviewers’ comments and concerns.

Reviewer #1: In this paper, the authors have proposed EEG based emotion recognition while applying cosmetic cream using deep learning. The work is interesting; however, it needs minor revisions in accordance with the following comments-

The introduction of this manuscript is very long. It must be concise. I am going to suggest how authors may shorten the introduction.

-First three paragraphs (1st paragraph, section 1.1, section 1.2) could be merged into one paragraph. Last sentence of first paragraph (In this study, meaningful results) should be cited properly.
**Answer: The first three paragraphs (1st paragraph, Section 1.1, Section 1.2) have been merged into one paragraph and duplicate content has been removed.**

- Section 1.3, 1.4, 1.5 contain very basic information. I think no need a detailed explanation of this information. Authors may use a small paragraph to present this information with proper citation.
**Answer: Sections 1.3 to 1.5 have been merged into first three paragraphs, and duplicate content has been deleted.**

-In section 1.6 and 1.7, the authors reviewed lots of related studies very comprehensively. These sections should also be concise.

**Answer: We searched a lot of literature related to EEG-based emotion recognition using machine learning and deep learning. In order to say that the data feature extraction method, deep learning model structure, and model validation method presented in this study are appropriate, we had to investigate and list the methods presented in many existing studies.**

-In the last paragraph, the authors should mention some research gaps based on the related study above. Then, the authors should mention their contributions to overcome those gaps. These contributions must be very specific, informative and not more than 4 points. Here, please avoid generalizations like- point 6 of section 1-8: “developing CNN models with various structures”.
**Answer: In the last paragraphs of 1-8, we mentioned the following contributions compared to previous studies. “Compared with previous studies, our study verified the model with a more thorough method (using LOSO CV, preventing overfitting by using validation dataset) in applying the deep learning method. In addition, a new attempt was made to recognize human emotions by presenting new stimuli.”**

**In order to avoid generalizations such as number 6 in Sections 1-8, "Developing CNN models with various structures" was changed to: “We compared the seven CNN models we proposed and found the best CNN structure.”**

-In section 3-2 (Data balancing), the authors performed data augmentation to balance the data. To explain the process, the authors made this statement- “That is, after randomly selecting data from a small number of classes for each subject, increasing the data and making the number of data equal between the two classes.” Is there any citation to support this statement? In the case of a random selection of sample/observation, if the majority of wrong sample/observations are selected, then the accuracy will be lower than actual. Conversely, if the majority of right sample/observations are selected, then the accuracy will be higher than the actual. The authors should clarify this issue.

**Answer: We have reordered Sections 3-3 (Windowing and reshaping) and Section 3-2 (Data balancing) to make things more clear to our readers about data balancing. Because in data balancing, data is the result after windowing and reshaping. In section 3-2 (data balancing), we introduced a paper (citing [79-80]) on random over-sampling. Since it was assumed that the same emotion would persist for the same stimuli for one subject, it is thought that the data in the randomly selected time window will be similar to each other.**

-In Figure 5, the authors merged time-frequency image of 19 electrodes in 5*5 matrix. That’s a great idea. However, if the number of the electrodes is different i.e., 5/8/30/60/64 or more, how the image will be merged? The authors should clarify this issue.

**Answer: If the number of electrodes is 5/8/30/60/64 or more, first make a square matrix with the largest number closest to the number of electrodes (e.g., 6x6 matrix when the number of electrodes is 30), and then, considering the distance between the electrodes, the near electrodes can be placed close to each other and distant electrodes can be placed farther away.**

-In the result and discussion, the author claimed that stacked CNN achieved the highest accuracy over other methods. It will be excellent if authors explain- for which properties of stacked CNN, it achieved the highest accuracy? Why other methods achieved comparatively lower accuracy? It is normal that the accuracy of stacked CNN is also very low as compared to the other EEG based application or EEG based emotion recognition. However, the authors should explain the reasons behind this lower accuracy.

**Answer: The reasons why stacked CNNs performed the best compared to other methods are as follows.**

**1. In the case of inception-like CNN and parallel band input CNN, the structure is somewhat more complicated than that of stack CNN, and there are more parameters to learn than stack CNN, so the accuracy may be low.**

**2. In case of single band input CNN, performance is considerably lower than that of 4band merged input CNN, probably because the amount of information of input data is reduced in single band.**

**The reason why it is low compared to other EEG-based applications or EEG-based emotion recognition is that the brain's mechanism for expressing emotions when applying the cream is different from the conventional mechanism for vision or smell, so we think the accuracy may be different. Additionally, while other studies have presented visual and olfactory stimuli from different people, in our study, signals such as muscle noise were mixed while subjects applied the cream directly, possibly resulting in reduced accuracy.**

-In pages 17 and 18, the authors mentioned the padding in the last column of this table. I suggest mentioning the number of padding for this experiment.

**Answer: We mentioned ‘padding: same’ in Tables 3 and 4, and explained about padding as follows below the table. “The padding type ‘same’ means that the output feature map has the same spatial dimensions as the input feature map. Zero padding is introduced to ensure that the input map has the same shape as the output map on all sides.”**

-In page 19-20 (Table 5), the authors should clarify why they used a specific Learning rate of 0.0002 and a maximum epoch of 300?

**Answer: When the optimizer is Adam, researchers usually set the learning rate to 0.001. However, in our study, we found that when the learning rate was 0.001, the validation loss value increased rapidly from 1 epoch. Therefore, it was important to learn slowly at the beginning because it was not possible to obtain the minimum value of the validation loss (the minimum value was obtained in a random state before training). Since the learning rate was very low, the learning progressed slowly, and the maximum epoch was set to 300 to check that the maximum and minimum point of validation accuracy and validation loss appear (see Figure 11).**

-The caption for Figure 4, 5, 11, 12, 13, and 14 should be concise.
**Answer: Each figure caption has been edited for conciseness.**

-Please improve the presentation of Figure 3, Figure 9 (just remove free space), Figure 14

**Answer: Figure 3 and Figure 14 has been improved. Figure 9: Removed free space.**

-English should be revised in any section of the paper because some typos are present throughout the manuscript and the used English should be polished by further proofreading.

**Answer: We have completed English proofreading by a professional English proofreading company. I am attaching the certificate (Certificate_of_editing-QPQKI_1_v3j6xrt_p7.pdf).**

---------------------------------------------------------------------------------------------------------------------------------------

Reviewer #2: The study aims to investigated different CNN architecture towards classifying cosmetic preferences. The following are my comments:

Introduction:
Quite lengthy, some parts could be merged and summarized. Consider trimming down the content and highlight the major findings of previous studies before describing the aim of the present investigation and how it addresses the gap in the body of knowledge
**Answer: The first three paragraphs (1st paragraph, Section 1.1, Section 1.2) have been merged into one paragraph and duplicates have been removed. Sections 1.3 to 1.5 were merged into first three paragraphs, and overlapping contents were deleted. The main results of the previous study were first emphasized, and then, the purpose of this study and how to address the gap between the knowledge system was explained in the final section (1.3 Motivation of our study) of introduction.**

Methods:
Data Balancing: Augmentation - was the selected data (small number of classes) replicated? It was not specifically mentioned how it was increased.

**Answer: We've reversed the order of sections 3.3(Windowing and reshaping) and 3.2 (Data balancing) to make it easier for readers to understand. Because in data balancing, data is a 5-second interval after windowing for each trial of each subject, so it would be good to mention data windowing first. Also, for readers to understand, paper (citing [79-80]) that was used and studied on random over-sampling in previous EEG-based analysis was additionally introduced.**

4.2.1 - Why only the inception model employed, since there are other transfer learning models available, i.e., VGG16 amongst others?
**Answer: We have never used a transfer learning model. The Inception-like model used in our paper is a much shallower model than the model published by Google. However, although the layer is shallow, the structure is similar to inception, so it was named inception-like model. In our study, it was not suitable to use a transfer model (i.e., VGG16, ResNet50, Google Inception model) with many parameters to learn due to the small number of data for our study.**

Table 4, how was the hyperparameters selected? was it optimised or is it merely heuristic?
**Answer: When the optimizer is Adam, researchers usually set the learning rate to 0.001. However, in our study, we found that when the learning rate was 0.001, the validation loss value increased rapidly from 1 epoch. Therefore, it was important to learn slowly at the beginning because it was not possible to obtain the minimum value of the validation loss (the minimum value was obtained in a random state before training). Since the learning rate was very low, the learning progressed slowly, and the maximum epoch was set to 300 to check that the maximum and minimum point of validation accuracy and validation loss appear (see Figure 11). The mini-batch size 128 was chosen to be within the limits of the computer's GPU memory.**

Results: Table 12 & 13, missing (%) symbol on the Y-axis.

**Answer: In Fig 12 and 13, the symbol (%) is displayed on the y-axis.**
